# Subchronic Toxicity and Effect of the Methanolic Extract of *Micromeria frivaldszkyana* (Degen) Velen on Cognition in Male Wistar Rats

**DOI:** 10.3390/plants14121837

**Published:** 2025-06-15

**Authors:** Elisaveta Apostolova, Kristina Stavrakeva, Vesela Kokova, Ivica Dimov, Mariya Choneva, Delyan Delev, Ilia Kostadinov, Iliya Bivolarski, Maria Koleva, Tsvetelina Mladenova, Krasimir Todorov, Anelia Bivolarska

**Affiliations:** 1 Department of Pharmacology, Toxicology, and Pharmacotherapy, Faculty of Pharmacy, Medical University of Plovdiv, Vasil Aprilov Str. 15A, 4002 Plovdiv, Bulgaria; kristina.stavrakeva@mu-plovdiv.bg (K.S.); vesela.kokova@mu-plovdiv.bg (V.K.); 2 Research Institute, Medical University of Plovdiv, 4002 Plovdiv, Bulgaria; iliya.kostadinov@mu-plovdiv.bg; 3 Department of Medical Biochemistry, Faculty of Pharmacy, Medical University of Plovdiv, Vasil Aprilov Str. 15A, 4002 Plovdiv, Bulgaria; ivica.dimov@mu-plovdiv.bg (I.D.); mariya.choneva@mu-plovdiv.bg (M.C.); anelia.bivolarska@mu-plovdiv.bg (A.B.); 4 Department of Pharmacology and Clinical Pharmacology, Faculty of Medicine, Medical University of Plovdiv, Vasil Aprilov Str. 15A, 4002 Plovdiv, Bulgaria; delyan.delev@mu-plovdiv.bg; 5 Department of General and Clinical Pathology, Faculty of Medicine, Medical University of Plovdiv, 4000 Plovdiv, Bulgaria; iliya.bivolarski@mu-plovdiv.bg (I.B.); mariya.koleva@mu-plovdiv.bg (M.K.); 6 Department of Botany and Biological Education, Faculty of Biology, University of Plovdiv “Paisii Hilendarski”, 24 Tsar Assen Str., 4000 Plovdiv, Bulgaria; cmladenova@uni-plovdiv.bg (T.M.); ktodorov@uni-plovdiv.bg (K.T.)

**Keywords:** subchronic application, biochemical markers, activity cage, shuttle box, passive memory, spatial and working memory

## Abstract

Recently, compounds of plant origin have been the focus of increased scientific interest. *Micromeria frivaldszkyana* is a rare species endemic to Bulgaria, whose biological activity remains unknown. This article aims to evaluate the subchronic toxicity of *Micromeria frivaldszkyana* methanolic extract and its effect on cognition in rats. Following 90 days of oral administration, a histopathological evaluation of brain, kidney, and liver tissues was conducted. Additionally, serum levels of total bilirubin (TB), conjugated bilirubin (CB), alanine aminotransferase (ALT), aspartate aminotransferase (AST), creatinine (CR), uric acid (UA), and urea (U) were measured. Cognitive function was studied after 7 d of treatment using activity cage test, along with tests for active memory, passive memory, anxiety, spatial and working memory, and explorative activity. The experiments showed no toxic effects of the extract in subchronic application. No adverse effects on brain function were observed after 14 days of treatment. While the extract increased the motor activity of the animals, it did not significantly improve the learning and memory processes. In conclusion, the methanolic extract of *Micromeria frivaldszkyana* in doses 250 and 500 mg/kg bw did not induce toxicity after 90-day treatment in rats. These doses did not significantly affect central nervous system (CNS) functions, although increased motor activity was observed after 14 days of treatment with the extract.

## 1. Introduction

Herbal medicines are available in a myriad of over-the-counter formulations, and their role in the global pharmaceutical market continues to expand [1]. Plant-derived compounds (such as pectin, baicalin, and curcumin) are widely utilized in the development of novel pharmaceutical formulations [2]. Essential oils containing monoterpenes (e.g., cineole, eugenol, limonene, citronellol, citronellal, camphor, and thymol) are commonly used as mosquito repellents [3]. Interest in plant-based remedies continues to grow due to their general safety and efficacy.

*Micromeria frivaldszkyana (M. frivaldszkyana),* Lamiaceae, is a rare plant species listed in the Red Data Book of the Republic of Bulgaria [4,5]. Scientific studies on *M. frivaldszkyana* are scarce, likely in relation to its limited natural habitat. The earliest research primarily focused on its phytochemical composition [6,7].

The antioxidant capacity was assessed using the DPPH radical scavenging assay, in which the methanolic extracts of *Micromeria dalmatica* and *Micromeria frivaldszkyana* demonstrated the most pronounced activity [7]. More recent studies have further highlighted both the antioxidant and antimicrobial properties of *M. frivaldszkyana* extracts. The antioxidant activity of the plant’s aerial parts was quantified using multiple assays, yielding the following results: DPPH—286.4 ± 10.43 mM TE/g, ABTS—358.4 ± 10.4 mM TE/g, FRAP—388.0 ± 32.4 mM TE/g, CUPRAC—905.6 ± 19.2 mM TE/g, ORAC—3250.5 ± 208.1 µmol TE/g, and HORAC—306.1 ± 23.5 µmol GAE/g. Notably, the ORAC value of *M. frivaldszkyana* exceeds that of many other Bulgarian medicinal plants, suggesting its potential as a potent natural antioxidant.

In terms of antimicrobial activity, the Gram-positive bacterium *Listeria monocytogenes* ATCC 19111 exhibited sensitivity to the *M. frivaldszkyana* extract, as evidenced by an inhibition zone of 9 mm and a minimum inhibitory concentration (MIC) of 10 mg/mL [8].

Other members of the Lamiaceae family, such as *Stachys germanica* (essential oil), also demonstrate antioxidant and anti-inflammatory activities, which are attributed to bioactive constituents such as bornyl acetate and camphor [9].

Plants from the genus *Micromeria* have a long history of use in folk medicine for treatment of cardiac diseases, pulmonary pathologies (asthma), and for their wound healing properties. Other indications include headache, cold, infections of the skin, etc. Scientific reports reveal antirheumatic, antiseptic, and CNS-stimulating properties of different species of the genus,; however, the information about the species *M. frivaldszkyana* remain limited [7,10,11,12].

Chemical investigations of *Micromeria fruticosa* have primarily focused on its volatile constituents [13]. High-performance liquid chromatography (HPLC) analysis of its methanolic extract identified ferulic acid, catechin, chrysin, and catechol as the predominant phenolic acids, while quercitrin and rutin were the major flavonoid glycosides. The aqueous extract of *M. fruticosa* has demonstrated a range of pharmacological activities, including anti-inflammatory, analgesic, antitumor, antioxidant, hepatoprotective, and gastroprotective effects [14].

Similarly, *Micromeria biflora* has been shown to possess anti-inflammatory, analgesic, and antipyretic properties, which are likely due to the high concentrations of caryophyllene oxide and β-eudesmol found in its essential oil [15].

Methanol and water extracts of *Micromeria myrtifolia* are characterized by high concentrations of rosmarinic, syringic, chlorogenic, caffeic, and protocatechuic acids, while the ethyl acetate extract predominantly contains rosmarinic acid and apigenin. Among the extracts, the aqueous extract exhibits the highest antioxidant activity, along with the greatest total phenolic and flavonoid content, followed by the methanolic extract [16].

The ethanolic extract of the aerial parts of *Micromeria croatica* has demonstrated hepatoprotective effects against carbon tetrachloride (CCl_4_)-induced liver injury and fibrosis. In a murine model of chemically induced hepatic intoxication, treatment with *M. croatica* significantly reduced oxidative stress, inflammation, and fibrogenesis [17].

In our previous study, UPLC-MS/MS analysis of methanolic extracts of *M. frivaldszkyana* led to the identification of 192 compounds. The most abundant secondary metabolites were phenolic acids and flavonoids, primarily in the form of flavonoid glycosides. The compounds present at the highest concentrations included linarin and its derivatives, chlorogenic acid, rutin, eupatorin, and rosmarinic acid [18].

Linarin has been shown to exert anti-inflammatory effects by reducing the levels of nitric oxide (NO), tumor necrosis factor-alpha (TNF-α), interleukin-6 (IL-6), and prostaglandin E2 (PGE2) in LPS-stimulated RAW264.7 macrophage cells [19]. It has also demonstrated anticholinesterase effects in both in vitro and ex vivo models. Acetylcholinesterase (AChE) activity was significantly decreased in two brain regions following intraperitoneal administration of linarin at different doses (35, 70, and 140 mg/kg) in mice [20].

Additionally, linarin exhibits hepatoprotective effects by significantly reducing the damage to liver structure, infiltration of inflammatory cells, elevated serum transaminases, and pro-inflammatory cytokines caused by CCl_4_. It also mitigated CCl_4_-induced oxidative stress by upregulating the expression of cytosolic Nrf2 (nuclear factor erythroid 2-related factor 2) and the stress-responsive protein heme oxygenase-1 (HO-1). Furthermore, linarin lowered the expression of toll-like receptor 4 (TLR-4) [21].

Chlorogenic acid also exhibits notable anti-inflammatory effects through modulation of key inflammatory signaling pathways. Specifically, chlorogenic acid has been shown to inhibit the growth of hemolytic *Streptococcus* and reduce the production of IL-6, TNF-α, and IL-1β, thereby suppressing the inflammatory response in mice infected with the pathogen [22]. In addition, in streptozotocin-induced diabetic rodent models, chlorogenic acid effectively decreased the expression of pro-inflammatory cytokines, including TNF-α and IL-6, contributing to the attenuation of systemic inflammation [23]. Furthermore, chlorogenic acid contributes to glycemic regulation by enhancing insulin sensitivity and lowering blood glucose levels [24] and has demonstrated protective effects against liver injury in various experimental models [25].

Rutin is recognized for its potent antioxidant capacity, protecting cells from oxidative stress. It also exhibits antidiabetic effects through modulation of glucose metabolism. Studies have documented the antihyperglycemic effects of rutin across a broad dosage range (5–100 mg/kg) and via various administration routes, including oral and intraperitoneal injection [26]. Additionally, anti-inflammatory activity has been reported for rutin and its glycosides in vitro [27]. Moreover, rutin has vasoprotective properties, strengthening capillary walls and reducing their permeability [28].

Eupatorin has shown cytotoxic activity against several human cancer cell lines [29] and anti-inflammatory effects, primarily through inhibition of key pro-inflammatory mediators [30].

Rosmarinic acid is known for a wide spectrum of biological activities, including antiviral, antibacterial, and anticancer effects, as well as robust antioxidant properties. It has also been associated with anti-aging, antidiabetic, cardioprotective, nephroprotective, antidepressant, and antiallergic effects [31]. Rosmarinic acid has demonstrated antinociceptive and anti-inflammatory properties, along with hepatoprotective activity in both in vitro and in vivo studies [32]. Additionally, it has been reported to inhibit acetylcholinesterase activity in rodent brain tissue and to reduce oxidative stress-induced neuronal damage [33].

Previously, metabolic composition of the extract was evaluated and its anti-inflammatory activity in rats was reported. Methanolic extract of *M. frivaldszkyana* was particularly rich in linarin and its derivatives, chlorogenic acid, rutin, eupatorin, and rosmarinic acid. Oral application of the extract for two weeks decreased the paw edema during the early phase of inflammation (1st to 3rd h after carrageenan injection) [18].

Liver pathologies are associated with alterations in hepatic enzyme levels, which facilitate the detection of hepatotoxicity through blood sample testing. The release of aspartate aminotransferase (AST) and alanine aminotransferase (ALT) enzymes into the bloodstream serves as an indicator of hepatocellular damage or alterations in liver cell membrane permeability. ALT is relatively more specific to hepatocytes, whereas AST is present at higher levels in various tissues, including the liver, muscle, and heart. Consequently, elevated ALT levels are more indicative of liver-specific damage, while increased AST may reflect injury in multiple tissue types. Unconjugated bilirubin is a pigmented substance primarily produced through the breakdown of heme by macrophages during the degradation of aged red blood cells. Damage to liver cells impairs their normal bilirubin excretion, resulting in elevated bilirubin levels in the blood. Historically, the ratio of unconjugated (indirect) to conjugated (direct) bilirubin has been used to distinguish between hepatic and extrahepatic causes of hyperbilirubinemia [34,35].

The objective of this study was to evaluate the subchronic toxicity of a methanolic extract of *M. frivaldszkyana* in rats. To achieve this, histopathological examinations of the brain, kidney, and liver were conducted, and key biochemical markers indicative of liver and kidney toxicity were measured in rat serum. Additionally, to assess the potential effects of the extract on central nervous system function, behavioral tests evaluating motor activity, memory (active and passive), and exploratory behavior were performed following 7 days of oral administration of the extract.

## 2. Results

### 2.1. Subchronic Toxicity—Histopathological Evaluation

During the 90 days of the subchronic toxicity study, no behavioral changes in either the control or the treated groups were detected. Additionally, no clinical signs of toxicity were observed in any of the animals.

**Figure 1:**
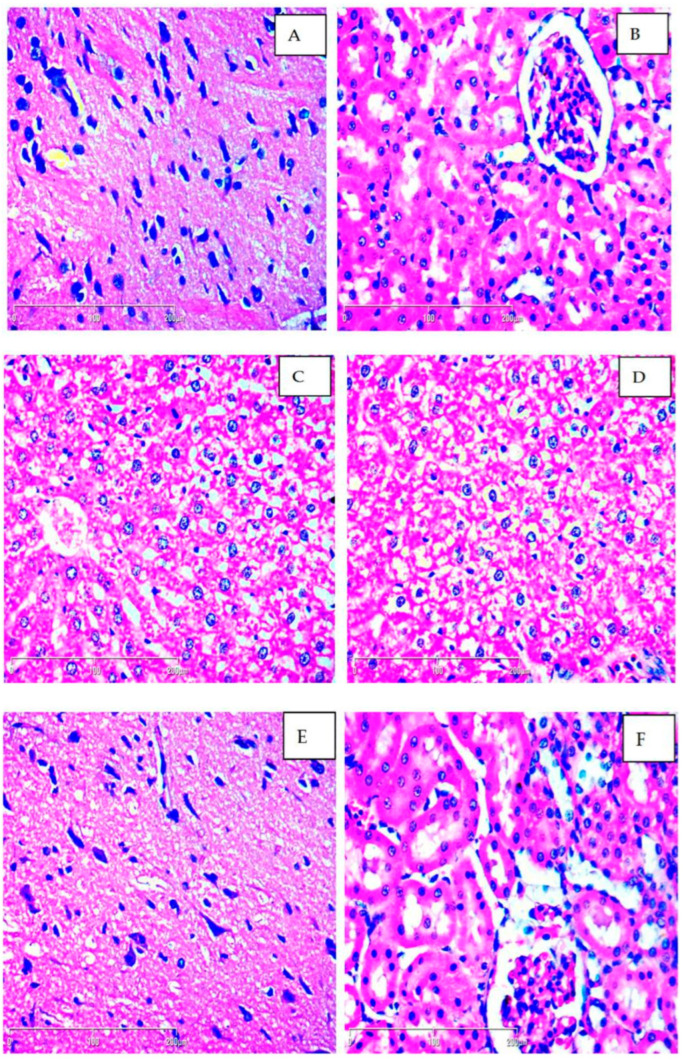
Histological examination of the brain (**A**), kidney (**B**), and liver (**C**) tissues in rats treated for 90 days with M. frivaldszkyana extract revealed preserved histological architecture in the brain and kidney and mild-to-moderate hepatic steatosis in the liver. Panels (**D**–**F**) show histological sections of the liver, brain, and kidney, respectively, from the control animals, exhibiting comparable histological features. The tissue sections were stained with hematoxylin and eosin (H&E) and examined at 400× magnification.

Macroscopic comparison of the liver, kidneys and brain between the control and treated animals revealed no differences in shape, size, color, or texture. Observation of the tissue samples did not reveal any significant differences between the control group and experimental groups (250 mg/kg and 500 mg/kg methanolic extract of *M. frivaldszkyana*). Across all groups, mild to moderate steatosis was observed (Grade 1 or 2 according to Roenigk classification), likely attributable to dietary factors. The structure of the brain and kidney tissues was normal and no major pathological lesions in organs were found (Figure 1).

### 2.2. Subchronic Toxicity—Biochemical Markers

#### 2.2.1. Liver Toxicity Markers

**Figure 2:**
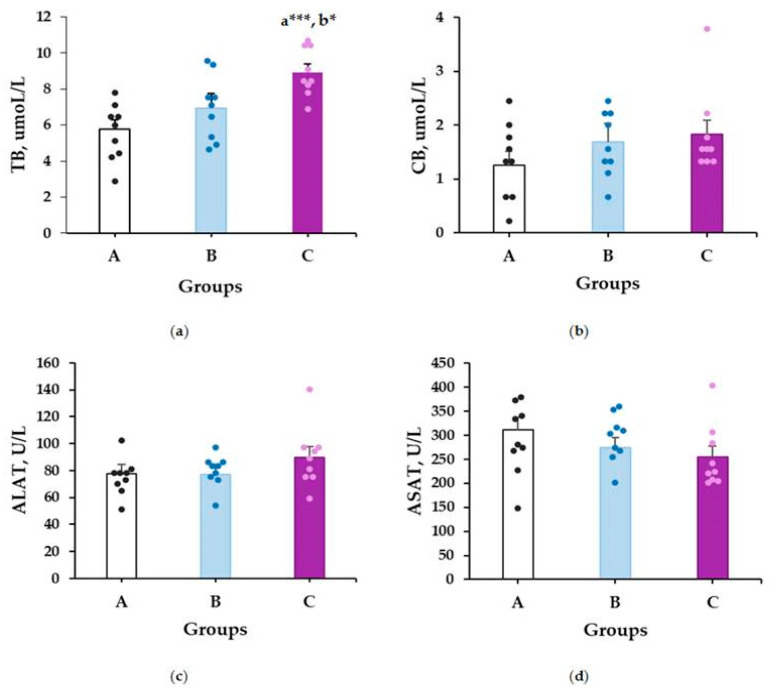
Serum concentrations of (**a**) total bilirubin (TB); (**b**) conjugated bilirubin (CB); (**c**) alanine aminotransferase (ALT); and (**d**) aspartate aminotransferase (AST). The data are presented as mean ± SEM along with individual raw values (*n* = 9). One-way ANOVA revealed significant differences between groups for the TB levels: *F*(2, 24) = 10.293, *p* = 0.001. No significant differences were observed for the other markers: CB: *F*(2, 24) = 1.140, *p* = 0.366; AST: *F*(2, 24) = 1.027, *p* = 0.373; ALT: *F*(2, 24) = 1.809, *p* = 0.185. Symbols: * indicates a significant difference compared to group B (*p* ≤ 0.05) and *** indicates significant differences versus group A (*p* ≤ 0.001), based on one-way ANOVA followed by Tukey’s post hoc test. A—control group (distilled water); B—experimental group, treated with 250 mg/kg *M. frivaldszkyana* methanolic extract; C—experimental group, treated with 500 mg/kg *M. frivaldszkyana* methanolic extract.

The data on the evaluated liver function markers are presented in Figure 2. According to the statistical analysis, treatment of the rats with methanolic extract of *M. frivaldszkyana* at a dose of 500 mg/kg for 90 days resulted in a significant increase in serum TB levels compared to the control group (8.94 ± 0.44 vs. 5.78 ± 0.51, *p* ≤ 0.001) (Figure 2a). Additionally, the TB levels in the 500 mg/kg group were significantly higher than those in the group treated with a twofold lower dose (8.94 ± 0.44 vs. 6.94 ± 0.82, *p* = 0.032) (Figure 2a). No statistically significant differences were observed in the remaining liver function markers (Figure 2b–d).

#### 2.2.2. Kidney Toxicity Markers

**Figure 3:**
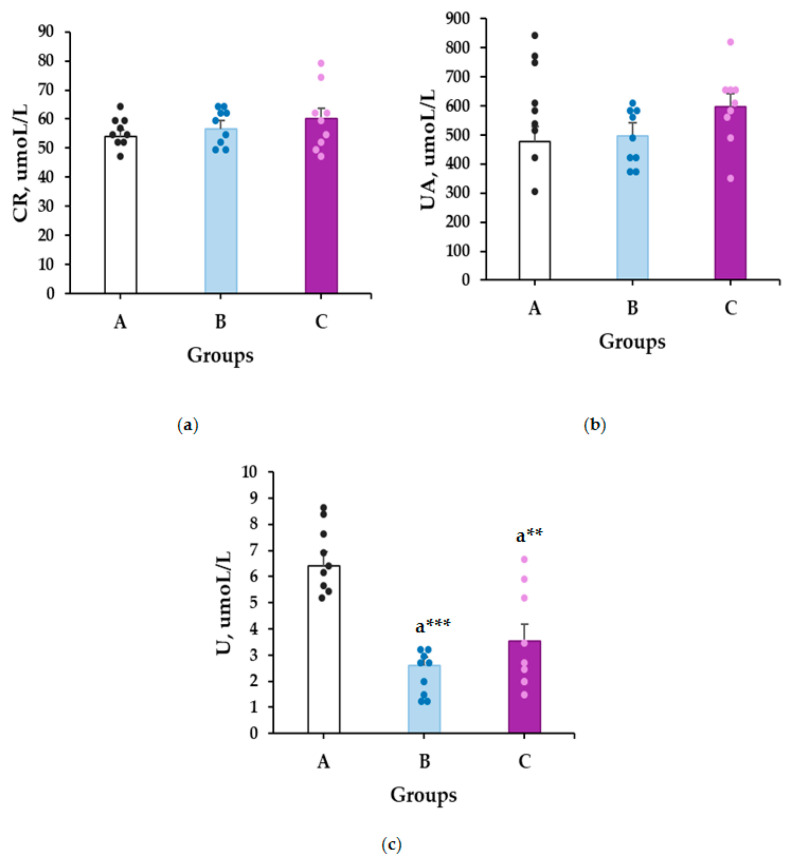
Serum concentrations of (**a**) creatinine (CR); (**b**) uric acid (UA); and (**c**) urea (U) concentration. The data are presented as mean ± SEM along with individual raw values (*n* = 9). One-way ANOVA revealed significant differences between groups for the U levels: *F*(2, 24) = 23.640, *p* = 0.000. No significant differences were observed for the other markers: UA: *F*(2, 24) = 1.746, *p* = 0.196; C: *F*(2, 24) = 0.717, *p* = 0.498. Symbols: ** indicates significant differences versus control group A (*p* ≤ 0.01); *** indicates significant differences versus control group A (*p* ≤ 0.001), based on one-way ANOVA followed by Tukey’s post hoc test. A—control group (distilled water); B—experimental group, treated with 250 mg/kg *M. frivaldszkyana* methanolic extract; C—experimental group, treated with 500 mg/kg *M. frivaldszkyana* methanolic extract.

The results for the kidney toxicity markers are presented in Figure 3. No statistically significant differences were observed in serum CR and UA levels among the rats (Figure 3a,b). However, as shown in Figure 3c, the 90-day treatment with *M. frivaldszkyana* methanolic extract at both 250 mg/kg and 500 mg/kg resulted in a significant reduction in serum urea levels compared to the control group (2.61 ± 0.3 vs. 6.41 ± 0.52, *p* ≤ 0.001; 3.53 ± 0.63 vs. 6.41 ± 0.52, *p* ≤ 0.001, respectively).

### 2.3. Effects on Cognitive Function

#### 2.3.1. Activity Cage

**Figure 4:**
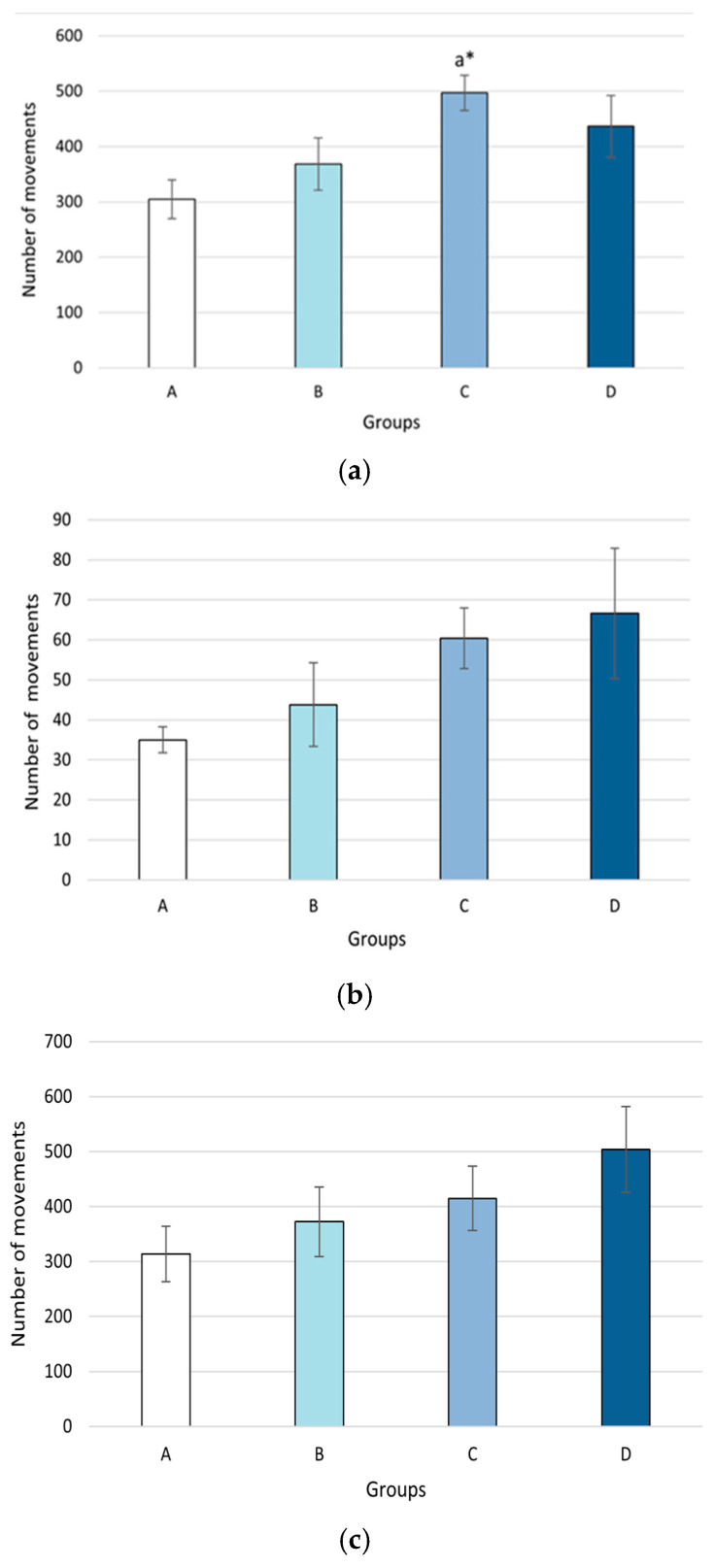

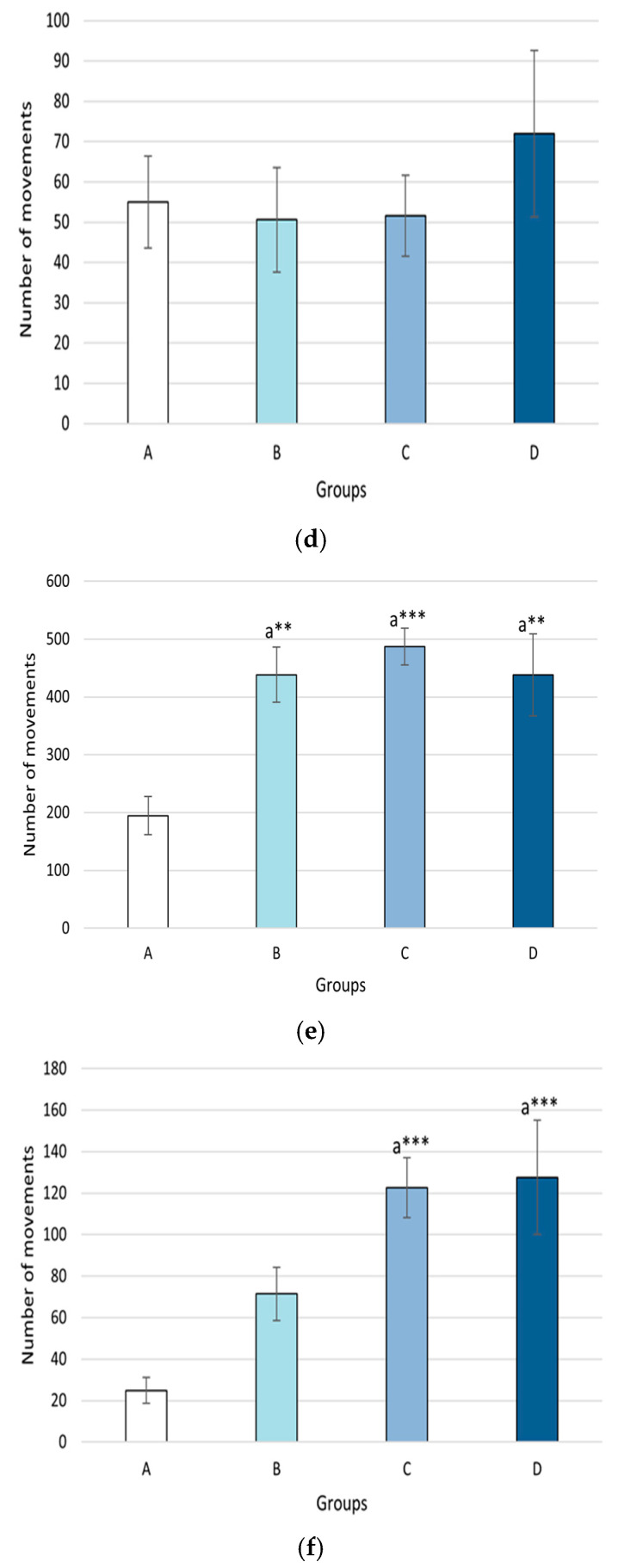
The effect of methanolic extract of *M. frivaldszkyana* on the number of horizontal movements on day 1 (**a**), day 7 (**c**), day 14 (**e**), and vertical movements on day 1 (**b**), day 7 (**d**), and day 14 (**f**) in rats. The data are presented as mean ± SEM (*n* = 8). One-way ANOVA showed significant differences in the horizontal activity between group C and the control group on day 1: *F*(3, 36) = 3.646, *p* = 0.018; group B and the control group: *F*(3, 36) = 7.384, *p* = 0.006 on day 14; group C and the control group: *F*(3, 36) = 7.384, *p* = 0.001 on day 14; group D and the control group: *F*(3, 36) = 7.384, *p* = 0.006 on day 14. Significant differences were detected in the vertical activity between groups C, D and the control group: *F*(2, 24) = 7.984, *p* = 0.001 on day 14. No significant differences were observed for the movements on the rest of the days: horizontal activity on day 7: *F*(3, 36) = 1.591; vertical activity on day 1: *F*(3, 36) = 1.928; vertical activity on day 7: *F*(3, 36) = 0.477. The symbol * indicates significant differences versus controls * (*p* < 0.05); ** (*p* < 0.01); *** (*p* ≤ 0.001). (One-way ANOVA, Tukey’s post hoc test). A—control group, treated with distilled water; B—experimental group, treated with 250 mg/kg *M. frivaldszkyana* methanolic extract; C—experimental group, treated with 400 mg/kg *M. frivaldszkyana* methanolic extract; D—experimental group, treated with 500 mg/kg *M. frivaldszkyana* methanolic extract.

The results of the study are presented in Figure 4. The group treated with the methanolic extract at a dose of 400 mg/kg showed a significant increase in horizontal movements on the first day of the study (496.6 ± 31.87 vs. 304.7 ± 35.0, *p* = 0.018). This trend is also observed in the horizontal movements on the 14th day, in the groups treated with methanolic extract at doses of 250, 400, and 500 mg/kg, showing an increase compared to the control group (438.3 ± 47.85 vs. 194.7 ± 33.17, *p* = 0.006; 486.8 ± 31.65 vs. 194.7 ± 33.17, *p* = 0.001; 437.7 ± 70.85 vs. 194.7 ± 33.17, *p* = 0.006). A significant increase in vertical activity was also observed in the groups treated with the methanolic extract at doses of 400 mg/kg and 500 mg/kg on the 14th day (122.6 ± 14.47 vs. 24.9 ± 6.18, *p* = 0.001; 127.6 ± 27.58 vs. 24.9 ± 6.18, *p* = 0.001).

#### 2.3.2. Two-Way Active Avoidance Test

Analysis of the number of conditioned and unconditioned responses (Table 1 and Table 2) after 14 days of extract treatment revealed no statistically significant differences between the experimental groups and the control group.

#### 2.3.3. Step-Through Passive Avoidance Test

**Figure 5:**
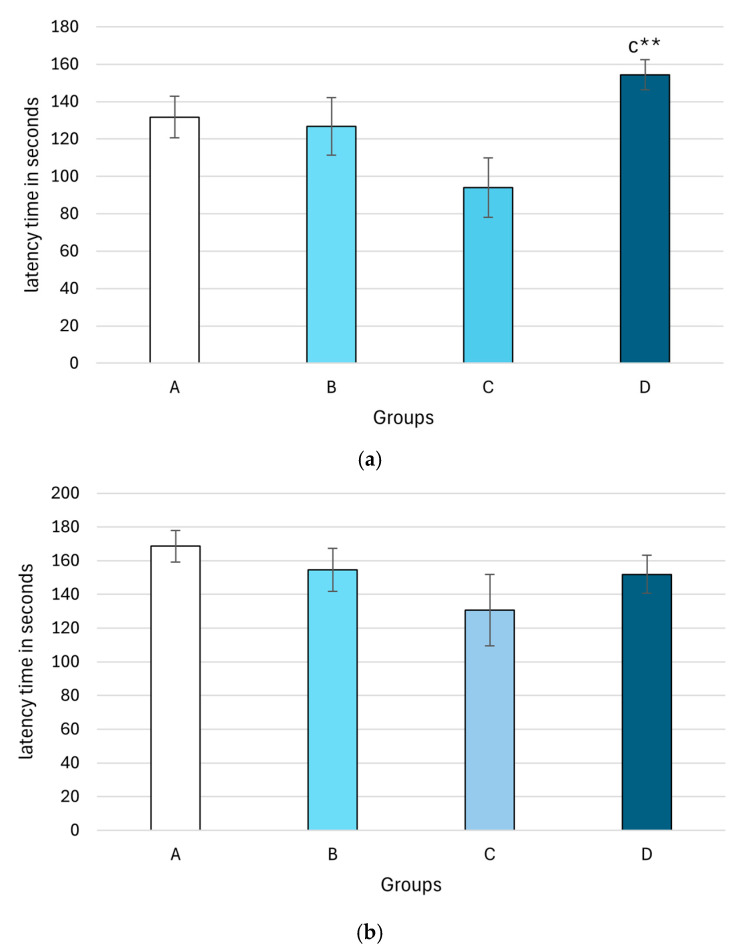
The effect of the methanolic extract from *M. frivaldszkyana* on the latency time in seconds on day 1 (**a**) and day 2 (**b**) in rats tested with the step-through apparatus. Note: The data are presented as mean ± SEM (*n* = 8). One-way ANOVA showed significant differences between group D and group C on day 1: F(3, 36) = 0.021, *p* = 0.01. No significant differences were observed on the second day: *F*(3, 36) = 0.332. The symbol ** indicates significant differences versus group C (*p* < 0.01). (One-way ANOVA, Tukey’s post hoc test). A—control group, treated with distilled water; B—experimental group, treated with 250 mg/kg *M. frivaldszkyana* methanolic extract; C—experimental group, treated with 400 mg/kg *M. frivaldszkyana* methanolic extract; D—experimental group, treated with 500 mg/kg *M. frivaldszkyana* methanolic extract.

Figure 5 shows an increase in the latency time in the group treated with methanolic extract in a dose of 500 mg/kg) compared to the group treated with methanolic extract with 400 mg/ kg bw (154.32 ± 8.14 vs. 93.96 ± 15.82, *p* = 0.01) on the first day of the training session.

#### 2.3.4. Step-Down Passive Avoidance Test

Table 3 shows no statistically significant difference in the latency time between the treated groups and the control group.

#### 2.3.5. New Object Recognition Test

The study revealed no statistically significant differences in the recognition index between the control and experimental groups (Table 4).

#### 2.3.6. Y-Maze

**Figure 6:**
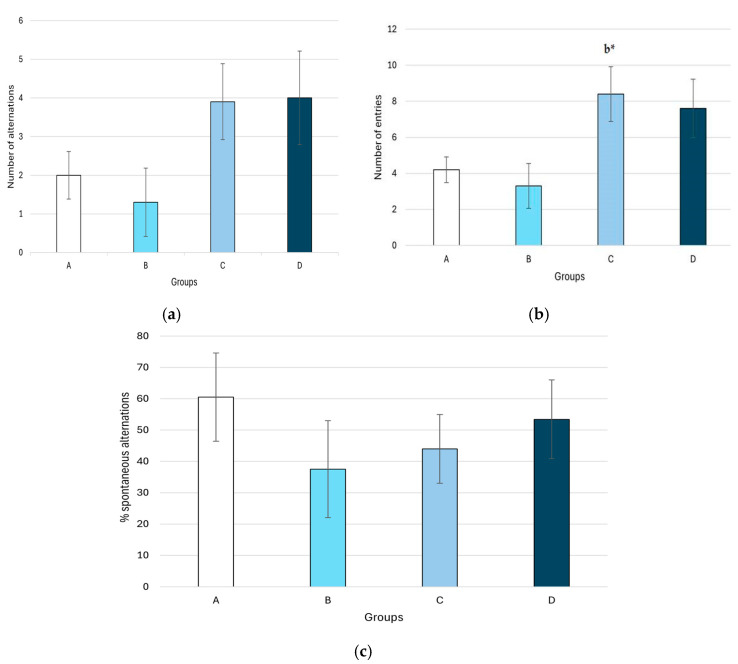
The effect of methanolic extract of *M. frivaldszkyana* on (**a**) the number of alternations, (**b**) the number of entries, and (**c**) the percentage of spontaneous alternations in the Y-maze test. *Note:* The data are presented as mean ± SEM (*n* = 8). One-way ANOVA revealed a significant difference in the number of entries between Group C and Group B: *F*(3, 36) = 0.023, *p* = 0.046. No significant differences were observed in the number of alternations: *F*(3, 36) = 0.123, or in the percentage of spontaneous alternations: *F*(3, 36) = 0.636. The symbol * indicates a significant difference compared to Group B (*p* < 0.05; One-way ANOVA with Tukey’s post hoc test). A—control group, treated with distilled water; B—experimental group, treated with 250 mg/kg *M. frivaldszkyana* methanolic extract; C—experimental group, treated with 400 mg/kg *M. frivaldszkyana* methanolic extract; D—experimental group, treated with 500 mg/kg *M. frivaldszkyana* methanolic extract.

The results of the spatial memory experiment (Figure 6) indicate a significantly higher number of entries in the group treated with the methanolic extract at a dose of 400 mg/kg compared to the group treated with 250 mg/kg body weight (8.4 ± 1.52 vs. 3.30 ± 1.24, *p* = 0.046).

#### 2.3.7. Elevated Plus Maze

According to Table 4, there is a dose-dependent trend toward an increased ratio of entries into the open arms relative to the total number of entries in all groups treated with the extract. However, a statistically significant difference compared to the control group was observed only in the group treated with 500 mg/kg of the methanolic extract (0.63 ± 0.08 vs. 0.32 ± 0.06; *p* = 0.032) (Table 5).

## 3. Discussion

During the histological examination of the liver, mild to moderate portal inflammation was observed across groups. These findings are not necessarily related to the application of the extract and may not indicate toxicity. They could be related to dietary factors, metabolic variation, or normal adaptative/compensatory changes in liver tissue. No major pathological hepatic lesions were observed. Biochemical test results supported the hypothesis of lack of toxicity, showing no changes in serum levels of AST and ALT after prolonged treatment with the extract (see Section 2.3. of *Results*). Furthermore, histological evaluation of kidney and brain tissue samples revealed normal architecture and no signs of toxicity. In general, these findings suggest that long-term exposure of rats to the methanolic extract of *M. frivaldszkyana* does not induce toxicity in the major internal organs. However, further in-depth analysis, including long-term studies combined with functional assessments, is necessary to confirm these findings.

Previously, we reported that the extract exhibited no acute toxicity in Wistar rats, when administered orally at doses up to 5000 mg/kg [18]. The current study expands the safety exploration of the extract, demonstrating no toxic effects after prolonged treatment. This conclusion is supported by pathological examination of liver, brain and kidney tissues. After 90 days of treatment with the methanolic extract of *M. frivaldszkyana*, no significant structural changes were observed in the major internal organs of the rats when compared to controls. Biochemical evaluation of serum levels of AST, ALT, and CB also showed no statistically significant differences between experimental and control groups. A statistically significant increase was observed only in total serum bilirubin levels in the group treated with 500 mg/kg of the methanolic extract. This finding can be attributed to an elevation in unconjugated bilirubin (UB), a key component of total serum bilirubin. Common causes of elevated UB include erythrocyte hemolysis or enhanced enterohepatic circulation, neither of which suggest direct hepatic damage [36].

The main serum markers of kidney function (uric acid and creatinine) showed similar levels in controls and the *M. frivaldszkyana* extract-treated groups. A decrease in blood urea nitrogen (BUN) levels was observed in the groups treated with 250 mg/kg and 500 mg/kg of the extract when compared to controls. These findings reveal a beneficial effect of the extract on the kidney function and lack of nephrotoxicity.

According to the authors’ knowledge, this is the first study on the effects of the *M. frivaldszkyana* methanolic extract on cognition in vivo. The information about other *Micromeria* species is also limited. Essential oil and the ethanolic extract of *Micromeria fruticosa* have shown CNS stimulating properties, while the methanolic extract of *Micromeria myrtifolia* (*M. myrtifolia*) exhibited antidepressant properties in mice [37,38]. However, in our experiments, such activities were not observed for the *M. frivaldszkyana* extract. These discrepancies may be attributed to differences in phytochemical compositions, experimental methodologies, or the animal models employed.

Küpeli et al., 2019 [37] reported antidepressant properties of rosmarinic acid, myricetin, apigenin, and naringenin. The methanolic extract of *M. frivaldszkyana* is predominantly rich in linarin, rutin and eupatorin, followed by apigenin and rosmarinic acid. Naringenin was present in small amounts, and myricetin was not detected as a secondary metabolite in this plant [18]. Neuroprotective properties are reported for apigenin and rosmarinic acid. Apigenin was also found effective in slowing the progression of Alzheimer’s disease [39,40].

Rosmarinic acid has been shown to exert neuroprotective effects in preclinical models of neurological disorders. In models of cholinergic dysfunction, it increases acetylcholine levels, potentially supporting cholinergic neurotransmission [41]. It also attenuates lipopolysaccharide (LPS)-induced cognitive impairment by restoring antioxidant homeostasis and reducing levels of pro-inflammatory cytokines. Enhanced spontaneous alternations are reported in the Y-maze test [42]. Moreover, rosmarinic acid has demonstrated a dose-dependent protective effect against alcohol-induced cognitive deficits in rodents, as evaluated using the passive avoidance learning test [43]. Its administration improves behavioral outcomes in experimental models of Parkinson’s disease and amyotrophic lateral sclerosis (ALS), suggesting broad neuroprotective activity [44,45].

In Alzheimer’s disease models induced by amyloid beta (Aβ1–42), rosmarinic acid ameliorates cognitive dysfunction. Aβ1–42 is known to cause neurotoxicity through elevation of intracellular calcium and disruption of synaptic transmission. Rosmarinic acid mitigates these effects, and behavioral assessments, including the elevated plus maze, support its anxiolytic and antidepressant potential—likely linked to its antioxidative properties [46].

Additionally, multiple in vivo studies have reported antidepressant-like effects of rosmarinic acid, attributed to its modulation of both cholinergic and monoaminergic neurotransmitter systems [47,48]. Its anxiolytic activity is thought to be mediated, at least in part, through inhibition of T-type calcium channels [49]. Rosmarinic acid has also been associated with the alleviation of post-traumatic stress disorder (PTSD)-like symptoms [50] and exhibits antiepileptic activity in rodent models [51].

The findings of this study suggest that the methanolic extract of *M. frivaldszkyana* is well tolerated at the tested doses in rats and may modulate motor behavior, as evidenced by the increased locomotor activity observed in the treated groups. These results highlight the potential applicability of *M. frivaldszkyana* extract as a candidate for further investigation in managing conditions characterized by reduced locomotion, such as fatigue or mild depression. Moreover, the extract did not produce significant alterations in cognitive parameters, suggesting potential for future research on its effects on cognition in murine models of neurodegenerative diseases. However, the absence of cognitive enhancement and the unclear mechanism of action indicate the need for further investigation into its underlying pharmacological pathways.

Future studies should also focus on identifying the active phytoconstituents responsible for the observed effects, elucidating their molecular targets, and evaluating the extract’s efficacy in models of liver toxicity.

This study has the following limitations: The subchronic toxicity testing was performed only on male Wistar rats, the outcome may be different in female animals or different rat strains and routes of administration. The histopathological examination following the 90-day treatment with the methanolic extract was limited to the liver, kidney, and brain tissues. Other organs (stomach, heart, and reproductive and endocrine organs) were not included in the study protocol. The elevated plus maze test was performed on naïve rats, and the outcome may be different in rats subjected to experimental model of stress/anxiety.

## 4. Materials and Methods

The experimental protocol was approved by the Bulgarian Food Safety Agency (permit number: 352/30 May 2023) and the Ethics Committee of the Medical University, Plovdiv, Bulgaria (protocol number: 6/5 October 2023). The ARRIVE guidelines, the EU Directive 2010/63/EU for animal experiments, and the relevant national and institutional rules and regulations were taken into account during this study. The plant material was collected after obtaining a permit from the Ministry of Environment and Water (permit number 996/9 August 2023).

### 4.1. Chemicals and Reagents

Methanol (≥99.8%, cat. No. 179337) was purchased from Merck SA (Darmstadt, Germany). Eosin Y (1% aqueous solution, BIOCARE Medical (Pacheco, CA, USA), cat. № 294/EOY-10-OT-2.5 L), formaldehyde 4% (10% neutral buffer, BIOCARE 310 Medical (Pacheco CA, USA), cat. № 294/FNB4-10 L), Histanol 100 (BIOCARE 310 Medical (Pacheco, CA, USA), cat. № 294/H100-5L), Histanol 95 (BIOCARE 310 Medical (Pacheco, CA, USA), cat. № 294/H95-5L), hematoxylin G3 (BIOCARE 310 Medical (Pacheco, CA, USA), cat. № 294/HEMG3-OT-2.5L), acetone (BIOCARE 310 Medical (Pacheco, CA, USA), cat. № 48/3413/5) and xylol (BIOCARE 310 Medical (Pacheco, CA, USA), cat. № 348/3410/20) were purchased from BIOCARE Medical (Pacheco, CA, USA).

### 4.2. Plant Material and Preparation of the Methanolic Extract

Aerial parts of *M. frivaldszkyana* (approximately 800 g fresh plant material) were collected during the vegetation period of 2023–2024 within the Bulgarka Nature Park, floristic region of Middle Stara Planina Mountain, as previously described [18]. A voucher specimen of *M. frivaldszkyana* was deposited in the herbarium of the Agricultural University in Plovdiv (SOA) under reference number 062648. The plant material was dried naturally for 10 days in a shaded area at room temperature (22 ± 2 °C). The plants were considered dry when their leaf stalks broke when bent and the petiole crumbled when crushed. After the drying period, the plants were finely ground to a powder with average particle size of less than 400 μm in a laboratory mill (GRINDOMIX GM200, RETSCH GmbH, Haan, Germany).

The methanolic extract was obtained by initial maceration of 10 g of powdered plant material in 70% methanol (1:10 *w*/*v*). The mixture was stirred continuously for 24 h at room temperature (22 ± 2 °C) in a light-protected flask. To enhance extraction efficiency, a triplicate ultrasonification was performed, consisting of 3 cycles of 15 min at 30 °C. After centrifugation at 6000× *g* for 15 min, the resulting supernatant was filtered through Whatman No. 1 filter paper. The same extraction procedure was repeated twice on the remaining plant material. The three extracts were combined, and the solvent was evaporated under reduced pressure using a rotary evaporator (Heidoplh, Schwabach, Germany) at 50 °C until complete dryness was achieved [18].

### 4.3. Animals

For the study, Male Wistar rats were used. The initial weight of the animals was 80–120 g for the subchronic toxicity testing and 85–160 g for the cognition experiments. All animals were housed under the following standard laboratory conditions: temperature: 22 °C ± 1 °C, humidity: 45%, 12:12 h light/dark cycle, access to food and water: *ad libitum.*

### 4.4. Subchronic Toxicity—Treatment

The study protocol was similar to the design described in open access manuscripts [52,53,54,55]. Briefly, the animals were randomly assigned into three groups (each group consists of nine animals, *n* = 9). Group A (control) received distilled water (0.1 mL/100 g bw), while groups B and C received oral water solutions of the dried methanolic extract (prepared as described in Section 4.2) in doses 250 mg/kg bw (group B) and 500 mg/kg bw (group C). All treatments were administered once daily via gastric tube for a period of 90 days. During this period, the animals were monitored every morning (between 8:00 and 10:00 a.m.) for mortality and observable signs of toxicity, including behavioral changes. Body weight was measured for each rat before the first application (day 1 of the experiment) and at 7-day intervals during the whole study. On the 89th day, the animals were fasted for 15 h prior to blood and organ collection. The blood samples were collected on day 90 of the experiment by cervical decapitation after intraperitoneal injection of 5 mg/kg bw Xylazine. The brain, liver, and kidneys were isolated from each euthanized animal and subjected to histopathological observation.

#### 4.4.1. Histopathological Evaluation

Immediately after collection, the organs were immersed in 10% neutral buffered formalin solution for fixation. The samples were then embedded in paraffin blocks. The complete procedure consists of the following steps:

1. The tissues were fixed in formalin solution for 24 h, rinsed thoroughly, and then placed in distilled water for 30 min to remove residual fixative.

2. The samples were dehydrated by sequential immersion in 95% and 100% ethyl alcohol (Histanol^®^ 100%). Initially, the tissues were submerged in 95% ethyl alcohol for 4–5 h, which was followed by 100% ethyl alcohol exposition with the same duration.

3. The dehydrated tissues were washed with acetone for 20 min, followed by xylol for 30 min. At the end, they were embedded in melted paraffin and molded into blocks. Each paraffin block was sectioned into 5 μm thick slices, and the sections were stained with haematoxylin–eosin before light microscope examination.

Histopathological assessment was conducted independently by two pathologists, blinded to the treatment of the groups. Morphological features of tissues from experimental groups were compared to the control group using Leica DM 500 (Leica Microsystems, Wetzlar, Germany) and Zeiss AXIO Scope A1 (2.5×, 5×, 10×, 20×, 40×, and 63×; Jena, Germany) microscopes [52,53,54].

The kidney samples were evaluated for signs of necrosis, apoptosis, inflammation and vascular alternations. The liver sections were examined for disruption of normal architecture, portal or lobular inflammation, sinusoidal dilatation and congestion, granuloma formation, degeneration, necrosis, and fat deposition. Brain tissue samples were searched for necrosis, inflammation, and vascular changes.

Renal tissue damage was categorized into five grades by utilizing a scale of 0 to 5 as described by Ahmed et al. [55]:

0 = normal histology;

1 = tubular epithelial cell degeneration, without significant necrosis/apoptosis;

2–5 = tubular epithelial cell necrosis/apoptosis, accompanied by other concomitant changes present in ˂25%, ˂50%, ˂75% and ˃75% of the tubules.

The hepatic tissue sections were categorized according to the Roenigk score (Table 6).

#### 4.4.2. Biochemical Markers

The blood samples (3–4 mL) were collected in tubes containing a coagulation accelerating agent (VACUTEST KIMA, Arzergrande, Italy). They were immediately refrigerated, and upon visible coagulation, the samples were centrifuged at 3000 rpm for 10 min at 4 °C in a refrigerated centrifuge (MPW-352R, Warsaw, Poland) [57]. The obtained serum was subjected to a spectrophotometric analysis of the following parameters: AST, ALT, TB, and CB—for the assessment of the liver function, and U, CR, UA—for the assessment of the kidney function. The analysis was performed using an Evolution 300 UV-Vis spectrophotometer (Thermo Fisher Scientific, Waltham, MA, US), in accordance with the manufacturer`s instructions provided with the commercial kits (Human, Wiesbaden, Germany)

### 4.5. Evaluation of Cognitive Function

Four groups of animals (each group consists of eight animals, *n* = 8) were formed via random allocation of rats. Group A (controls) received orally distilled water (0.1 mL/100 g bw), while groups 2, 3, and 4 received water solutions of the dried methanolic extract orally (described in Section 4.2) in doses of 250 mg/kg bw (group B), 400 mg/kg bw (group C), and 500 mg/kg bw (group D). During all tests, the devices were cleaned with 90% ethyl alcohol after each rat to eliminate its odor before the next animal is placed in the apparatus.

Previously, we evaluated the acute toxicity of the extract and registered no toxic effects after a single oral application of the extract in doses up to 5000 mg/kg bw [18]. Based on these results, we estimated the doses for further evaluation of the effects of the extract: 250 and 500 mg/kg bw. According to Hanafy et al. [58], the doses suitable for further evaluation of the pharmacological effects of extracts of natural origin are 1/10 and 1/20 of the estimated LD50. We added the intermediate dose 400 mg/kg to obtain more detailed data on the potential effect of the extract.

**Figure 7:**
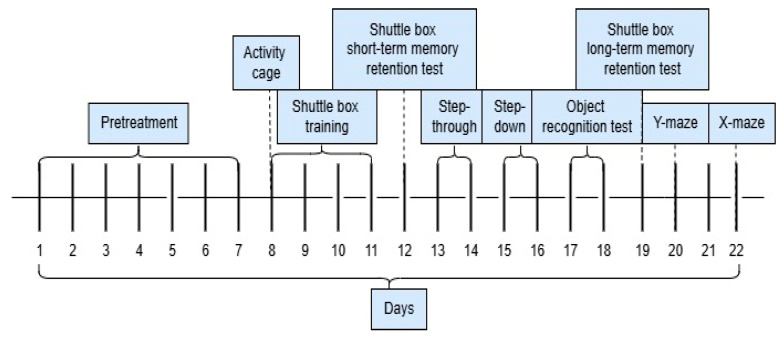
Sequence of the cognitive tests.

The experimental flow of the cognition tests is shown in Figure 7.

#### 4.5.1. Locomotor Activity Test

After 7 days of treatment, locomotor activity of the rats was assessed using the activity cage apparatus (Ugo Basile, Gemonio, Italy), as described previously [59]. Briefly, on the 8th day 30 min after the oral application of the extract, each animal was placed individually in the apparatus and was allowed to explore the new environment for 5 min. The number of horizontal and vertical movements of each rat was recorded.

#### 4.5.2. Shuttle-Box Active Avoidance Test

The active learning ability of the rats was tested using a fully automated shuttle-box apparatus (UgoBasile, Gemonio, Italy), according to the protocol reported by Mihaylova et al. [60]. Briefly, the rats were subjected to a training session for 4 consecutive days, with 30 trials per day, separated by 12 s intervals. Each trial included consecutive conditional stimuli (light and sound 670 Hz, 70 dB) with duration 6 sec and an unconditional stimulus (electrical foot shock 0.4 mA) for 3 s. On the 5th day the short-term memory was evaluated, and on the 12th day the long-term memory (without electrical stimuli) was tested. The first training session was initiated after locomotor activity testing on day 8 of the experiment (day 8 of the experiment corresponds to day 1 of the active avoidance test).

The following parameters were registered: number of avoidances (conditioned responses) and number of escapes (unconditioned responses).

#### 4.5.3. Step-Through Passive Avoidance Test

The passive learning ability of the rats was tested in a step-through apparatus (UgoBasile, Gemonio, Italy). The device consists of two compartments (one dark and one light), connected via a door. The floor of the dark chamber consists of metal wires, which allows the delivery of electrical shock. The protocol described by Rezaie et al. [61] was employed. Initially, the rat was allowed to explore the new environment. The animal was placed in the light chamber for 1 min with the door open, which allowed unrestrained movements between the two boxes. During the training sessions, the animals were placed again in the light box, however the door was closed. After 7 s, the door opened and remained open for the following 12 s. If during this period the rat had entered the dark chamber, the door closed and mild electric shock was delivered through the floor metal mesh (0.4 mA, 3 s). If the rat remained in the light compartment for 180 consecutive seconds, it was removed from the device.

The step-through passive avoidance test was performed on day 13 and 14 of the experiment. On day 13, three training sessions were conducted with intervals of 1 h. Long-term memory was assessed twenty-four hours after the last training session (day 14 of the experiment). The protocol was the same as in the training session; however, no electrical shock was delivered.

#### 4.5.4. Step-Down Passive Avoidance Test

Non-spatial long-term memory was tested in a step-down apparatus (UgoBasile, Gemonio, Italy) following the method of Ramalho et al. [62]. Initially, 3 training sessions with intervals of 1 h were performed, and 24 h after the last training session, the long-term memory was evaluated. A training session included placement of the rat on a vertically vibrating platform in the apparatus. Stepping down with all 3 or 4 paws onto the metal mesh floor triggered an electric shock (0.4 mA) for 3 s. If the animal remained on the platform for 1 min, it was removed from the chamber. Long-term memory was assessed by recording the latency time for the animal to step down without delivering a foot shock. The cut-off time was 300 s.

The training sessions were conducted on day 15 of the experiment and the long-term memory test was performed on day 16.

#### 4.5.5. New Object Recognition Test

Recognition memory was tested in activity cage apparatus (UgoBasile, Gemonio, Italy) on days 17 and 18 of the cognition experimental protocol according to the method, described by Lissner et al. [63]. On day 17, each rat was individually placed in the device and allowed to explore freely the new environment for 5 min (habituation phase), then was returned to its home cage. Twenty-four hours later (day 18), each rat was placed in the same apparatus again, this time with two identical objects positioned in the opposite corners of the chamber. The rats were allowed to explore them for 5 min (familiarization phase). The new object recognition test was performed 90 min later. The conditions were the same, except that one of the objects was replaced by a new one (unfamiliar to the rat). Again, the rat was allowed to explore the objects for 5 min, while the time for exploration of each object was recorded. The recognition index (RI) is calculated by Equation (1):

(1) $$RI=\frac{M_{1}}{{(M}_{1}+M_{0})},$$

where

M_0_—time spent for exploration of the familiar object;

M_1_—time spent for exploration of the unfamiliar object.

#### 4.5.6. Y-Maze Test

Short-term spatial memory was evaluated by Y-maze test, as described by Ghafarimoghadam et al. [64]. The test was performed using a Y-shaped Plexiglas device, which consists of three perpendicular arms, each labeled as A, B or C. Arms are 40 cm in length, 15 cm in width, and 30 cm in height. The test was performed on day 20 of the experiment (Figure 7). Each rat was placed at the center of the maze and its movements were observed for 8 min. The sequence of the arm entry was recorded. An entry was counted when the rat fully entered an arm (A, B, or C) with all four paws. If a rat entered all three arms consecutively, an alternation was recorded. To identify spontaneous alternation, an overlapping technique was used. For example, a pattern of A–C–B–C–A consists of two alternations [65]. The percentage of the spontaneous alternations (SA%) is calculated using the following Equation (2):

(2) $$SA\%=\left(\frac{number\;of\;SA}{total\;number\;of\;entries-2}\right)\times 100.$$

#### 4.5.7. Elevated Plus Maze Test

Anxiety-like behavior in rats was tested with elevated plus maze, following the protocol of Knight et al. [66]. Each rat was placed at the center of the apparatus facing an open arm and observed for 5 min. The following parameters were recorded: (1) number of entries and time spent in the open arms; (2) number of entries and time spent in the closed arms of the maze; (3) total number of entries in the arms of the apparatus; (4) open arm entries/total arm entries ratio.

### 4.6. Statistical Analysis

The statistical analysis was conducted using SPSS statistical software, version 17.0 (IBM, Armonk, NY, USA). All data are presented as mean ± SEM. The mean values were compared using one-way analysis of variance (ANOVA) and Tukey’s post hoc test. Values of *p* ≤ 0.05 were considered statistically significant.

## 5. Conclusions

This study aimed to evaluate the subchronic toxicity and neurobehavioral effects of the *M. frivaldszkyana* methanolic extract in male Wistar rats. The results support the following conclusions:

1. Subchronic toxicity: Oral administration of the *M. frivaldszkyana* methanolic extract at doses of 250 mg/kg and 500 mg/kg over a 90-day period did not induce signs of systemic toxicity. Histopathological examination of liver, kidney, and brain tissues revealed no major pathological changes, and biochemical markers of liver and kidney function remained within normal limits, indicating good tolerability of the extract at the tested doses.

2. Cognitive and motor function: Across multiple behavioral paradigms, the extract did not significantly enhance active or passive memory in treated animals. This suggests that, under the conditions of this study, the *M. frivaldszkyana* extract does not exert a substantial effect on cognitive performance in healthy rats. A dose-dependent increase in locomotor activity was observed after 7 and 14 days of treatment, as measured in the activity cage test. This indicates a potential stimulatory effect of the extract on motor function.

In conclusion, the methanolic extract of *M. frivaldszkyana* appears to be safe at the tested doses in male rats and may influence locomotor activity without altering cognitive performance. These findings justify further research to identify the active compounds, explore the mechanisms of action underlying the observed motor effects, and assess the extract’s efficacy and safety in animal models of neurodegenerative disorders.

## Figures and Tables

**Table 1:** The effect of methanolic extract from *M. frivaldszkyana* on the number of conditioned responses. Note: The values are presented as group mean ± SEM (*n* = 8). No significant differences (*p* ˃ 0.05) were observed in the number of avoidances on day 1: *F*(3, 36) = 1.073; day 2: *F*(3, 36) = 0.221; day 3: *F*(3, 36) = 0.447; day 4: *F*(3, 36) = 1.052; day 5: *F*(3, 36) = 0.944; day 12: *F*(3, 36) = 1.071 (One-way ANOVA, Tukey’s post hoc test). A—control group, treated with distilled water; B—experimental group, treated with 250 mg/kg *M. frivaldszkyana* methanolic extract; C—experimental group, treated with 400 mg/kg *M. frivaldszkyana* methanolic extract; D—experimental group, treated with 500 mg/kg *M. frivaldszkyana* methanolic extract.

Day Groups	1	2	3	4	5	12
A	1.30 ± 0.34	1.80 ± 0.89	4.10 ± 1.48	4.10 ± 1.63	5.90 ± 1.68	5.30 ± 1.82
B	0.50 ± 0.23	0.50 ± 0.23	1.70 ± 0.77	2.00 ± 0.99	3.50 ± 1.51	3.30 ± 1.34
C	1.10 ± 0.41	0.50 ± 0.31	1.90 ± 1.15	3.10 ± 1.39	3.30 ± 1.72	4.80 ± 2.14
D	0.50 ± 0.17	0.50 ± 0.41	0.70 ± 0.39	3.00 ± 1.43	4.50 ± 1.67	5.20 ± 1.76

**Table 2:** The effect of methanolic extract from *M. frivaldszkyana* on the number of unconditioned responses. The values are presented as group means ± SEM (*n* = 8). No significant differences (*p* ˃ 0.05) were observed in the number of escapes on day 1: *F*(3, 36) = 0.373; day 2: *F*(3, 36) = 0.881; day 3: *F*(3, 36) = 0.721; day 4: *F*(3, 36) = 0.381; day 5: F(3, 36) = 0.429; day 12: *F*(3, 36) = 0.374 (One-way ANOVA, Tukey’s post hoc test). A—control group, treated with distilled water; B—experimental group, treated with 250 mg/kg *M. frivaldszkyana* methanolic extract; C—experimental group, treated with 400 mg/kg *M. frivaldszkyana* methanolic extract; D—experimental group, treated with 500 mg/kg *M. frivaldszkyana* methanolic extract.

Day Groups	1	2	3	4	5	12
A	12.5 ± 2.82	10.30 ± 2.72	13.2 ± 2.97	14.7 ± 3.12	15.1 ± 3.04	16.7 ± 3.11
B	11.3 ± 2.83	8.9 ± 2.41	9.1 ± 2.63	8.5 ± 2.83	9.2 ± 2.46	10.6 ± 2.38
C	17.2 ± 2.86	11.7 ± 3.33	11.9 ± 2.88	8.8 ± 2.98	12.5 ± 2.47	11.2 ± 3.02
D	16.8 ± 3.03	8.9 ± 2.87	10.1 ± 2.43	10.1 ± 2.17	10.9 ± 2.31	12.0 ± 2.08

**Table 3:** The effect of methanolic extract of *M. frivaldszkyana* on the latency time in seconds in rats, examined with the Step-down apparatus. Note: Mean ± SEM are presented; *n* = 8. No significant differences (*p* ˃ 0.05) were observed in the latency time on day 1: *F*(3, 36) = 0.690; day 2: *F*(3, 36) = 0.232 (One-way ANOVA, Tukey’s post hoc test). A—control group, treated with distilled water; B—experimental group, treated with 250 mg/kg *M. frivaldszkyana* methanolic extract; C—experimental group, treated with 400 mg/kg *M. frivaldszkyana* methanolic extract; D—experimental group, treated with 500 mg/kg *M. frivaldszkyana* methanolic extract.

Day Groups	1	2
A	17.96 ± 3.31	25.77 ± 5.76
B	23.12 ± 3.81	36.73 ± 6.71
C	23.42 ± 3.99	38.71 ± 5.26
D	24.61 ± 5.41	41.63 ± 4.74

**Table 4:** The effect of methanolic extract of *M. frivaldszkyana* in a new object recognition test. Note: *Note:* The data are presented as mean ± SEM; *n* = 8. No significant differences (*p* > 0.05) were observed in the time spent exploring the novel object *F*(3, 36) = 0.149; recognition index, *F*(3, 36) = 0.262 (One-way ANOVA, Tukey’s post hoc test). A—control group, treated with distilled water; B—experimental group, treated with 250 mg/kg *M. frivaldszkyana* methanolic extract; C—experimental group, treated with 400 mg/kg *M. frivaldszkyana* methanolic extract; D—experimental group, treated with 500 mg/kg *M. frivaldszkyana* methanolic extract.

Groups	Seconds Spent Studying the New Object	Recognition Index
A	3.40 ± 0.62	0.55 ± 0.08
B	11.30 ± 4.29	0.58 ± 0.08
C	18.30 ± 7.78	0.56 ± 0.07
D	16.70 ± 4.06	0.73 ± 0.06

**Table 5:** Effect of methanolic extract of *M. frivaldszkyana* in the elevated plus maze test. *Note:* The data are presented as mean ± SEM (*n* = 8). One-way ANOVA revealed a significant difference in the ratio of open arm entries to total entries between Group D and the control group: *F*(3, 36) = 0.046, *p* = 0.032. No significant differences were observed in the time spent in the open or closed arms: *F*(3, 36) = 0.253; number of entries into the open arms: *F*(3, 36) = 0.270; or total number of entries: *F*(3, 36) = 0.499. The symbol * indicates a significant difference compared to the control group (*p* < 0.05; One-way ANOVA with Tukey’s post hoc test). A—control group, treated with distilled water; B—experimental group, treated with 250 mg/kg *M. frivaldszkyana* methanolic extract; C—experimental group, treated with 400 mg/kg *M. frivaldszkyana* methanolic extract; D—experimental group, treated with 500 mg/kg *M. frivaldszkyana* methanolic extract.

Groups	Seconds Spent in the Open Arms	Seconds Spent in the Closed Arms	Number of Entries into the Open Arms	Total Number of Entries	Ratio of the Number of Entries into the Open Arms to the Total Number of Entries
A	142.00 ± 30.16	158.00 ± 30.16	1.70 ± 0.52	3.90 ± 0.98	0.32 ± 0.06
B	106.70 ± 23.07	193.30 ± 23.07	2.00 ± 0.54	4.70 ± 1.17	0.41 ± 0.07
C	104.70 ± 25.89	195.30 ± 25.89	2.70 ± 0.47	5.90 ± 1.06	0.45 ± 0.07
D	67.30 ± 22.67	232.70 ± 22.67	3.30 ± 0.86	6.40 ± 1.72	0.63 ± 0.08 *

**Table 6:** Roenigk classification (adopted from Berends et al. [56]).

Grade	Accumulation of Fats	Nuclear Pleomorphism	Fibrosis	Necrosis/Inflammation
1	Mild or none	Mild or none	None	With or without mild portal inflammation
2	Moderate or severe	Moderate or severe	None	Moderate or severe portal inflammation
3a	With or without	With or without	Mild (fibrosis extending into acini)	With or without
3b	With or without	With or without	Moderate or severe	With or without
4	With or without	With or without	Cirrhosis	With or without

Grade 1—mild toxicity; Grade 2—moderate toxicity; Grade 3a—severe toxicity; Grade 3b or 4—indication for discontinuation of the treatment.

## Data Availability

The data presented in this study is available on request from the corresponding author.

## Abbreviations

The following abbreviations are used in this manuscript:

TB	total bilirubin
CB	conjugated bilirubin
AST	aspartate aminotransferase
ALT	alanine aminotransferase
CR	creatinine
UA	uric acid
U	urea
